# Team science in interdisciplinary health professions education research: a multi-institutional case study

**DOI:** 10.1007/s10459-024-10393-5

**Published:** 2024-11-16

**Authors:** Peggy Gesing, Joni Tornwall, Violet Kulo, Sarah McBrien, Thuha Hoang, Hyun-Jin Jun, Amanda Burbage, Yuane Jia, Christina Cestone

**Affiliations:** 1 Macon and Joan Brock Virginia Health Sciences at Old Dominion University EVMS School of Health Professions, P.O. Box 1980, Norfolk, VA 23501-1980 USA; 2 The Ohio State University, College of Nursing, Columbus, OH USA; 3 University of Maryland Baltimore, School of Graduate Studies, Baltimore, MD USA; 4 University of Nebraska Medical Center, College of Allied Health Professions, Omaha, NE USA; 5 Department of Physical Therapy, Louisiana State University Health Sciences Center, New Orleans, LA USA; 6 Rutgers, The State University of New Jersey, School of Health Professions, Newark, NJ USA

**Keywords:** Interdisciplinary collaboration, Team science, Health professions, Social learning, Social capital, Human capital

## Abstract

The interdisciplinary nature of health professions education requires exploration of problems by interdisciplinary research teams. Drawing on the Dynamics of Cross-disciplinary Research Development conceptual framework, this study explores the processes and experiences of an interdisciplinary research team, identifying elements of successful collaboration. Case study data were collected from a team of nine researchers from six universities. Data included meeting minutes and researcher reflections. Researcher reflection and meeting minute themes converged within the framework constructs: Contexts and inputs, social learning processes, social capital outcomes, and knowledge and human capital outcomes. This study provides a blueprint for interdisciplinary researchers indicating that successful collaboration starts with transparent, distributed leadership. The convergence of data illustrates how social learning processes, social capital outcomes, and knowledge and human capital outcomes facilitate research team outcomes.

**Supplementary Information:**

The online version contains supplementary material available at 10.1007/s10459-024-10393-5.

## Introduction

Health professions education (HPE) research is fundamentally interdisciplinary; therefore, exploration of problems in health education requires an interdisciplinary research team (O’Sullivan et al., 2010). Typically, interdisciplinary collaboration takes place through various stages of a project with researchers contributing diverse scientific knowledge drawn from different disciplinary and professional backgrounds in healthcare (Albert et al., 2020a, 2020b). The terms “interdisciplinary” and “interprofessional” are often used interchangeably in health professions education, but they are not always synonymous. In the context of health professions education research, “interprofessional” typically refers to research conducted by collaborating *healthcare professionals* (e.g., medical practitioners) whereas “interdisciplinary” refers to research conducted by teams composed of individuals from diverse *academic disciplines,* combining healthcare practitioners with academic researchers, and social scientists who integrate diverse disciplinary expertise to solve multifaceted problems that are beyond the scope of a single healthcare discipline or profession (Albert et al., 2020a, 2020b). Unfortunately, this has led to a gap in interdisciplinary and theory-based health professions education research. Therefore, the purpose of this post hoc case study was to explore the processes and experiences of members of an interdisciplinary HPE research team, each of whom are authors of this manuscript. The team members used the Dynamics of Cross-disciplinary Research Development framework to identify the elements that contributed to successful team science collaboration outputs: Social learning processes, social capital outcomes, and knowledge and human capital outcomes (Carr et al., 2018).

## Background

Interdisciplinary research that crosses disciplinary boundaries to integrate ideas and interprofessional practices produces meaningful changes in the way healthcare education is delivered (Fulmer & Gaines, 2014). Individual academic disciplines often have their own research methods, theories, and bodies of knowledge including anatomy, nursing, public health, physical therapy, and HPE (Krishnan, 2009; Ten Cate, 2021). Interdisciplinary research is theory-based and process-informed, and can be useful to address complex issues through questioning, gaining insights, and creating a comprehensive understanding of a problem (Menken & Keestra, 2016; Repko & Szostak, 2020). Interdisciplinary studies, integrate the elements of individual disciplines to evolve and include simple and complex structures, affecting institutional changes, including teaching and learning and ultimately gaining more encompassing perspectives (Klein & Newll, 1997).

Teamwork within interdisciplinary research is crucial to solving complex healthcare education problems, producing positive outcomes for dissemination of knowledge, access to resources, network development, and career advancement that would be otherwise inaccessible to individual researchers who work in isolation (Green & Johnson, 2015). Interdisciplinary research teams have better outcomes including greater productivity, scientific impact, and number of publications compared to less diverse teams and researchers working within disciplinary or professional boundaries (Hall et al., 2012; Jeong & Choi, 2015; Larivière et al., 2015). They are aware of the specificity of their academic discipline, value the expertise of team members, and accept co-responsibility across the disciplines for answering questions important to their study (Sell et al., 2022). They are more closely linked to others in research networks, participate in more collaborative activities, have more collaborators, produce more creative publications, and obtain more institutional resources than researchers on teams with a single disciplinary focus (Hall et al., 2018).

Interdisciplinary team performance is enhanced by development of models and conceptual frameworks across academic disciplines and healthcare professions (Fiore & Wiltshire, 2016; Paletz et al., 2013). Yet, teams face challenges including developing a common mission and shared processes, engaging in true mutual learning at the cost of difficulty or conflict, measuring and making tangible progress, balancing external demands, and a need for broad-based understanding of interdisciplinary team research beyond simply making efforts (McMurtry et al., 2012).Clarifying and describing the elements of successful interdisciplinary team research may provide a blueprint for future researchers that addresses these challenges as they establish their own interdisciplinary teams and engage in collaborative team research.

## Conceptual framework

This study utilized the Dynamics of Cross-disciplinary Research Development conceptual framework to provide a model that represents the principles, mechanisms, and processes involved in the evolution and progress of interdisciplinary research spanning multiple disciplines (Carr et al., 2018). It serves as a guide for a comprehensive understanding of how various professional fields influence each other, leading to the emergence of novel ideas, methodologies, and discoveries that transcend the boundaries of individual disciplines. This framework helps researchers and scholars navigate the complexities of interdisciplinary work, fostering collaboration, innovation, and knowledge integration across diverse fields of study (Carr et al., 2018).

The framework includes four constructs: (1) contexts and inputs, (2) social learning processes, (3) social capital outcomes, and (4) knowledge and human capital outcomes. The first construct acknowledges that the context in which a project operates significantly influences its processes and subsequent achievements (Stokols et al., 2008). A history of successful collaboration among researchers is likely to enhance their motivation and ability to collaborate further, thereby leading to more favorable outcomes (Borrego & Newswander, 2008). Context may include team size, group composition, physical setting (Carr et al., 2018; Stokols et al., 2003) and institutional support (Ponomariov & Boardman, 2010). The ideal team size and composition are influenced by the team objectives and the environmental contexts in which they operate (Stokols et al., 2008).

Social learning processes emphasize the need for a change in understanding at the individual level (intrapersonal) and its extension to broader social units (interpersonal), facilitated by social interactions within a network (Reed et al., 2010). A researcher’s capacity to adapt to evolving circumstances and embrace new perspectives is an essential element for successful collaboration during team cohesion building (Okhuysen, 2001). Individuals who prioritize collaboration, endorse a culture of sharing, and adopt an interdisciplinary ethos are aptly suited for interdisciplinary team science (Stokols et al., 2003). Intrapersonal values such as commitment, accountability, and a passion for achieving collaborative objectives further contribute to the potential success of interdisciplinary efforts (Stokols et al., 2008).

Social capital outcomes rely on interaction, connectivity, shared values and understanding, and trust (Carr et al., 2018). The convergence of diverse researchers fosters information exchange and yields new insights for each team member, leading to the generation of more innovative ideas and the development of shared understandings (Hibbert et al., 2016; Pyrko et al., 2017). In interdisciplinary programs and projects, a shared understanding in which diverse viewpoints are acknowledged, is considered a pivotal intermediary outcome. Effective processes that promote learning, interdisciplinary research practices, and interaction are seen as catalysts for the development of social capital, bolstering activities aimed at achieving shared goals, such as joint publications (Pretty, 2003).

Knowledge and human capital outcomes can vary based on the objectives of the participants. Individual goals may include developing professional networks, publishing in leading journals, or creating knowledge that benefits society at large. By integrating researchers’ objectives, research teams create knowledge and human capital outcomes that satisfy the needs of individuals on the team while contributing new knowledge (Carr et al., 2018).

The burgeoning interest in interdisciplinary collaboration has spurred a body of empirical research examining the intricacies of interdisciplinary teamwork within diverse academic and professional contexts. Determining the most suitable criteria to assess the success of interdisciplinary team science initiatives relies on how the fundamental elements of team performance and the critical aspects of interdisciplinary cooperation are defined (Stokols et al., 2008). Drawing on the Dynamics of Cross-disciplinary Research Development as its conceptual framework, this case study explores the processes and experiences of members of an interdisciplinary HPE research team using the following research questions:

RQ1: How do researchers on an interdisciplinary team navigate team dynamics to achieve collaborative research goals?

RQ2: How do researchers perceive their roles and contributions as members of an interdisciplinary research team?

## Methodology

Case study methodology was utilized to investigate the dynamics of a small research team in a real-world interdisciplinary context relying on multiple sources of converging evidence (Yin, 2018). Case study methodology was chosen to examine the contemporary event, e.g. collaborative research of an interdisciplinary team where the team behaviors could not be manipulated (Yin, 2018). This methodology was chosen over an ethnographic case study because process and personal experience, rather than culture, were used as a lens of focus (Merriam & Tisdell, 2016). The unit of analysis for this case is a small research team each of whom are authors of this manuscript, bounded by a research study conducted between April 2021 and March 2023.

Ethical approval was sought by the researchers and deemed unnecessary by Eastern Virginia Medical School’s Human Subjects’ Protection Program & IRB Office due to the researcher/team member dual role. Nonetheless, ethical considerations to address power dynamics, consent to participate, and trustworthiness in data analysis were made.

## Participants aka team members

This study explored the processes and experiences of an interdisciplinary team of nine researchers from six universities who collaborated on a multi-institutional, interdisciplinary HPE research project. The team science research project initially conducted by the team involved developing, distributing, and validating the Learning Modality Change Community of Inquiry and Self-efficacy scale to measure student experiences with the rapid transition to online learning during the COVID-19 pandemic (Jia et al., 2023). The nine original team members took on the dual role of researchers and participants for the current study of the team members’ experiences.

Participants in this case study are referred to as ‘team members’ throughout this manuscript. Team members’ interdisciplinary educational experience spanned health professions, education and instructional design, and research methodology. Most team members were unknown to each other and had various levels of expertise on the original topic of interest prior to joining the research project. Detailed analysis of team members’ CVs provided background information about the team member participants. Table1 includes the team members’ backgrounds and unique identifiers.

**Table 1:** Team members

Team member number	Team role*	Campus setting**	R1 class**	Academic rank	Industry/focus area
1	Lit review*	Midsize City	N	Assistant Professor	Health Prof Ed
2	Lit review	Large City	Y	Associate Professor	Health Prof Ed/PA
3	Lit review	Large City	Y	Associate Professor	Faculty Dev./Health Prof Ed
4	Lit review	Large City	N	Assistant Professor	Health Prof Ed/Assessment
5	Project lead, IRB	Midsize City	N	Assistant Professor	Health Prof Ed
6	IRB*	Large City	N	Assistant Professor	Physical Therapy/Health Prof Ed
7	IRB	Large City	Y	Associate Clinical Professor	Assessment/Nursing Ed
8	Scale dev	Large City	Y	Assistant Professor	Health Prof Ed/Methodology
9	Project lead, Scale dev.*	Large City	N	Assistant Professor	Methodology/Stats

*Indicates sub-team lead

**Team members’ institutional affiliations were identified using the National Center for Education Statistics (NCES) College Navigator to identify the institutions’ type and campus setting

Team members had a range of publication experience, and most had engaged in research and publication with at least one or more colleagues (Table 2). Analysis of the team member experience levels with various research processes using the Team Member Reflection Protocol (Appendix A) found that team members indicated they had a moderate amount of experience with quantitative and educational research and the IRB process, as well as survey design, team research, and team leadership. Most team members had almost no experience with interinstitutional and interdisciplinary research.

**Table 2:** Aggregate publication metrics

Publication category	Average	Range	Percentage of total publications
Total publications	18.00	4–35	
Total peer-reviewed publications	16.78	3–34	93.21%
Publications as sole author	0.33	0–2	.19%
Publications as first author	5.89	1–12	32.72%
Publications with two authors	4.78	0–15	93.83%
Publication with three or more authors	12.11	2–25	67.28%

## Setting

**Fig. 1:**
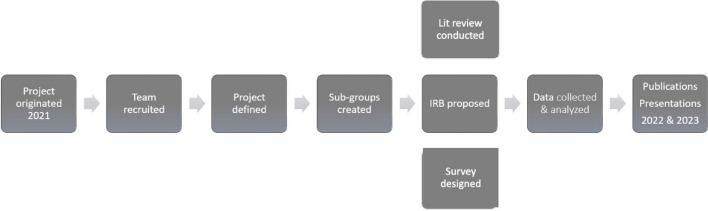
Timeline and process for the interdisciplinary collaboration

The original research in which the interdisciplinary team was formed was initiated by two members of American Educational Research Association (AERA) Division I, Education in the Professions. The two project leaders defined the research project goal, created a timeline, and identified the need for additional collaborators, leading to recruitment of nine additional team members from the division. Two team members dropped out in the first month, with the final group of nine team members meeting in May 2021 to refine the interprofessional research collaboration purpose. The team met monthly on the Zoom virtual meeting platform between April 2021 and March 2023, across three U.S. time zones and six states. Researchers used online collaboration tools including Microsoft OneDrive and OneNote. The team engaged in distributed leadership with sub-groups responsible for review of related literature, IRB proposal submission, and scale development. The distributed leadership model alleviated power dynamics, with team members contributing their strengths to the team effort. Team members successfully collaborated to publish three manuscripts (Burbage et al., 2022; Jia et al., 2023; Jun et al., 2024) and engage in three international conference presentations. Figure 1 provides a process map for the research project.

Figure 1 *Team Research Process Map*

## Data collection and analysis

Case study data sources include meeting notes and team member reflections. Consent to participate was discussed openly in research meetings and confirmed by voluntary contribution of a completed reflection. A protocol (see Appendix A) developed by team members 3 and 5 guided reflection data collection. Several trustworthiness measures were adopted to enhance credibility and quality of results and interpretation of findings including data triangulation and member checking. Data triangulation occurred by collecting and comparing data from multiple sources, including meeting notes and team member reflections, to identify consistent themes and patterns. This approach helped ensure the credibility and robustness of the findings by cross-verifying information from different perspectives. Ethical considerations were addressed by the use of multiple coders who reviewed blinded documents during analysis. Data were coded and converged to identify supported themes and patterns.

### Meeting minute data

Meeting minute data were used to answer RQ1: How do researchers on an interdisciplinary team navigate team dynamics to achieve collaborative research goals?

Minutes from 25 meetings which included shared decisions, priorities, future action items, and links to meeting recordings were reviewed and coded by team members 2 and 6. Meeting minutes of the first meeting were independently reviewed to generate first-cycle codes using descriptive coding. The remaining meeting minutes were coded independently using the first-cycle codes and additional codes were identified given that different topics were discussed during the meetings. Pattern coding was used for second-cycle coding to categorize the initial codes into sub-themes from which categorical themes were derived (Saldaña, 2021). Meeting minute themes included commitment as demonstrated by meeting attendance, roles and responsibilities, and progress and outcomes.

#### Theme #1: Commitment

The research team set up a standing monthly meeting with additional meetings scheduled occasionally as needed. A majority of team members (six to nine) attended almost all of the meetings. Excluding the first discussion meeting between the two original members, 18 meetings (75%) had six to nine attendees, and six meetings (25%) had three to five attendees. In addition, the sub-teams consisting of two to four members each met outside of the monthly team meetings to work on their assigned tasks. This high level of meeting attendance showed the commitment that team members had to the research project.

#### Theme #2: roles and responsibilities

A variety of responsibilities, such as drafting recruitment emails, conducting data analysis, manuscript writing and editing, and identifying journals for research dissemination, were handled by team members as needed. Project leaders facilitated meetings, assigned action items, and communicated project deadlines.

Members self-selected into sub-teams with sub-team leaders coordinating completion of assigned tasks. The IRB writing sub-team was responsible for drafting and submitting the IRB proposal for approval at four of the six institutions represented by researchers. IRB approval was not required by two of the six institutions represented. The literature review sub-team was responsible for identifying theoretical frameworks and providing an overview of related research. The survey design sub-team identified relevant validated scales and drafted survey questions. Sub-teams reported out at full-team meetings where consensus was required regarding project specifics like framework choice and survey question design.

#### Theme #3: progress and outcomes

Project status, including sub-team updates and next steps were discussed at each meeting. Final outcomes for research dissemination including publications and conference proposals were debated and agreed upon by the team. All major decisions were made with team consensus to ensure shared understanding and decision-making**.**

## Researcher reflections

Researcher reflection data were used to answer RQ2: How do researchers perceive their roles and contributions as members of an interdisciplinary research team?

Reflection data collection followed the reflection protocol (Appendix A) with each team member providing a written reflection based on the provided prompts. Written reflections were chosen over interviews to provide each team member ample time to reflect on their experiences. The reflection protocol was chosen over a survey because it allowed for open ended responses where team members provided answers in their own words capturing thick, rich descriptions of the unique personal experiences of each team member. This led to unexpected insights that could not be found with predefined survey choices.

The reflection protocol was designed by team members 3 and 5 and was reviewed and approved by the entire team prior to individual completion. It guided each team member through reflection of their motivations for joining the team, the barriers and challenges faced while on the team, the outcomes of being on the team, and practical advice for future research teams.

Reflections were de-identified and stored in a shared folder where they were visible only to the team members (3, 5, and 7) tasked with analyzing the data. Two team members (3 & 5) each reviewed one reflection to identify shared first-cycle codes. Once first-cycle codes were agreed upon, all reflections were deductively analyzed by team members 5 & 7 who identified quotes to support each first-cycle code. First-cycle codes and quotes from all reflections were reviewed and compared by the two team members to ensure agreement.

Second-cycle coding was conducted using ChatGPT 3.5, a form of generative AI that can generate and summarize text (ChatGPT, 2023). ChatGPT was used as a “third coder” to confirm first-cycle codes and identify second-cycle codes. All team members consented to the use of generative AI in the reflection data coding process.

The prompt, “What are the common categories across the following input?” was given to ChatGPT followed by de-identified team member quotes organized by first-cycle code. ChatGPT results were critically evaluated by team members 5 and 7 for accuracy, duplication, and alignment with the research questions. Line by line review of ChatGPT output identified convergence, contradiction, and redundancies among the first-cycle codes leading to refinement of codes and identification of themes and sub-themes (see Table 3). Example quotes were identified by team members 5 and 7 for each theme. Themes were then converged, and redundant codes were eliminated. Theme to quote correspondence was reviewed and agreed upon by researchers tasked with analyzing data. Four themes were identified in the reflection data: Leadership, motivation and benefits, collegiality, and challenges. Two to five sub-themes were identified within each theme (Table 3).

**Table 3:** Reflection Themes

#	Theme	Sub-themes
1	Leadership	Leadership & project management
		Team culture
2	Motivation & Benefits	Networking & collaboration
		Researching a new phenomenon
		Skill development
		Interinstitutional/interdisciplinary publication
		Promotion
3	Collegiality	Positive team experience
		Common values
		Diverse & unique contributions
4	Challenges	Time and personal obligations
		Team dynamics
		Multi-site logistics-IRB

### Theme #1: leadership

The leadership theme included two sub-themes: Leadership and project management, and team culture.

A majority (7/9) of team members cited team leadership as a reason for success and a reason for staying on the team including the “commitment of the leadership” and communication, “keeping minutes and documenting all discussions.” One team member stated, “I stayed, and I continue to stay because of strong leadership” from the team leader “but also, I see other members of the team having leadership roles, even if they aren’t formal roles.” This refers to a distributed leadership model where tasks were distributed to three sub-groups: IRB, literature review, and survey design, each with its own group leader. “Distributed leadership was one of the secret recipes for our success.”

Communication among team members was considered a strength of the team, with leadership facilitating monthly meetings, maintaining meeting notes, and communicating after and between meetings. “Good communication among team members within the larger group and subgroup is paramount, this happened during the meetings and via email so even when one was unable to attend a meeting, they knew the tasks at hand.” Setting monthly meetings at the start of the project encouraged team members to meet milestones and make progress. “We had monthly meetings, and it really did feel that from month to month we moved the needle at least just a little bit in terms of the project completion.”

Strong team culture was also cited as a strength by a majority of team members. The culture led to addressing disagreements and tough conversations with positive outcomes. “I have watched members of our group navigate conversations in a professional, productive manner.” The strong culture allowed for the preservation of “the dignity of all members while still holding them accountable.” Those who were in leadership roles acknowledged the challenge of being inclusive while also moving forward. Using tactics like identifying and playing to team member strengths as well as setting deadlines and sending reminders were successful leadership strategies.

### Theme #2: motivation & benefits

The motivation and benefits theme included five sub-themes: Networking and collaboration, researching new phenomena, skill development, interinstitutional/interdisciplinary publication, and promotion.

Networking and collaboration were motivators for most team members, with one team member describing development of professional friendships that led to a network of “colleagues that I can meet up with at conferences or can reach out to when I need professional help or advice.” Motivations related to networking and collaboration were found to differ by experience level, with several early career scholars recognizing that relationships would be “fruitful,” increase productivity, and “might lead to other future projects.” More experienced researchers claimed that working with the group provided “identity affirmation” within the context of health professions education where educational researchers are often the outliers. “It became just a really good team experience and that is why I kept working with everybody long term. When you find good people who work hard you stick with them.”

Team members also identified researching a new phenomenon, the impacts of COVID-19 on medical and health professions education, as a motivator, calling it a “once in a lifetime type of educational event.” Studying COVID-19 was a way of “contributing to the literature in education regarding a phenomenon that was unique, experienced by all of us, and not yet fully researched or yet understood in terms of its impact.” It was also a way to explore what was happening outside their institutions. “I didn’t really know what was happening with other campuses. It was illuminating and this study seemed to add to what we were learning.”

Team members were motivated by and benefited from skill development, and the opportunity to “learn from others,” “gain new experiences/skills in educational research,” and “learn more about other areas of health professions education… and about other institutions.” They valued learning new skills “I always learned something from each meeting and each member.” Finally, team members learned new skills by observing leadership styles and approaches. “I would like to emulate [a specific leadership skill]” was a commonly expressed desire in identifying opportunities for professional growth.

It also showed me where I could improve and that’s really valuable because it’s not always easy to identify your own gaps in knowledge or in research abilities but seeing others shine in their own abilities and being impressed or proud of them gives me something to recognize as something I can improve on or strive towards.

Team members were also motivated by the prospect of publication, with some specifically focused on the value of interinstitutional research and publication. One team member stated that publishing with team members from larger research institutions “brought acknowledgement or prestige.” Others appreciated the opportunity to get “multiple scholarly products from the project.” Finally, nearly half of all team members acknowledged that interinstitutional research would add value to their CV and dossier for promotion. The experience resulted in the desire to continue working together "I would like for our team to continue conducting research on niche topics in HPE teaching and learning so we can elevate our profiles among other HPE researchers.”

### Theme #3: collegiality

The collegiality theme included three sub-themes: Positive team experience, common values, and diverse and unique contributions.

Collegiality was recognized by all team members as an important element of success: “This is where I learned the power of team research.” They valued the “productive conversations about the project that expanded to topics about work, family, life, etc.,” with one team member stating, “It was great to work on a team like that and not have anybody’s ego get in the way as we made decisions.” The experience reaffirmed the value of team science for some “This specific teamwork has made me certain that team research can work, and it really can be better than research done in a silo.”

Most team members cited common values as key to the collegiality with one team member specifically identifying “cooperation, collaboration, accountability, mutual respect and trust, and integrity as some of values that I found in this team.” Another team member stated that “the work ethic and dedication of each member made the project move along smoothly”. Mutual respect motivated members but also allowed for support from the team.

At any time when I felt frustrated or that I couldn’t do all things I recognized that I would not want to let any of the team down and that I could just acknowledge whatever was happening with my workload and actually rely on the team to make space for these things.

Diverse and unique contributions were considered valuable by a majority of team members. Some cited the “opportunity to learn from other scholars who have different perspectives, experiences, and wisdom” as a benefit of the collaboration and indicated that unique contributions resulted in success. “This project definitely changed the way I think about team research because I did not give enough credit to the value of having different experiences institutions and skill sets on the team. I underestimated the unique contribution that any one person could provide.”

### Theme #4: challenges

The theme related to challenges included three sub-themes: Time and personal obligations, team dynamics, and multi-site logistics.

Time and personal obligations were identified by all team members as a challenge. Most time challenges resulted from the project being only a small part of each team members’ workload: “This type of project is counted as 10% of my time for my own scholarly activities, but it is not the only project that I have involved [sic] with” making “fitting the project in with the other day-to-day tasks I was doing” a challenge. This was acknowledged to be particularly challenging for faculty in health professions programs with heavy teaching and service loads “they do not have enough time for commitment to scholarly activities especially for this type of project (outside of daily job requirement).” Timeliness of the research topic was also a factor: “Because COVID was sort of winding down, we did have a sense of urgency that we needed to get the research out sooner.”

Several team members identified team dynamics as a challenge in that “developing working relationships” took time because few team members had worked together prior to the project. This period of relationship development led to initial challenges conceptualizing the project and “just narrowing the scope of our work.” In addition, one team member observed that that the relatively large number (9) of members in the group was a challenge. “In this case having so many people involved in the project was useful but there were times when getting everybody together or on the same idea page was a challenge so a smaller group of three to five might be more workable.”

Multi-site logistics were identified as a challenge during the IRB process because “each institution has different IRB protocol format required”. Researchers were also frustrated by low survey response rates at some institutions where faculty and students are saturated with surveys.

## Convergence results

Convergence of case study data involves the process of integrating multiple sources of data to draw a more comprehensive and coherent understanding of the case being studied. Viewing data from meeting minutes and reflections through the Dynamics of Cross-disciplinary Research Development conceptual framework (Carr et al., 2018) led to identification of convergent themes. Table 4 illustrates the Social Learning Processes, Social Capital Outcomes, and Knowledge and Human Capital Outcomes of the interdisciplinary research process in which the research team engaged.

**Table 4:** Data convergence: dynamics of Cross-disciplinary research development conceptual framework (Carr et al., 2018)

Social learning processes	Data type	Social capital outcomes	Data type	Knowledge & human capital outcomes	Data type
Team culture	RD	Leadership & project management	RD	Researching a new phenomenon	RD
Skill development	RD	Networking & collaboration	RD	Interinstitutional/interdisciplinary publication	RD
Positive team experience	RD	Promotion	RD	Multi-site logistics	RD
Diverse & unique contributions	RD	Common values	RD	Progress and outcomes	MD
Time & personal obligations	RD	Commitment	MD		
Team dynamics	RD	Roles & responsibilities	MD		
Progress and outcomes	MD				

^*^Data type: Reflection data (RD), Meeting minute data (MD)

Learning to navigate team culture and dynamics, develop skills, and maintain project progress while managing other obligations demonstrates how team members engaged in social learning processes. Individual learning occurred and interdisciplinary research practices were utilized as team members with diverse backgrounds interacted. Social capital outcomes were evidenced by the ability to interact within the team which was facilitated by leadership and project management. Shared values and commitment resulted in shared understanding, while networking and collaboration led to interpersonal connectivity. Knowledge and human capital outcomes were evidenced by ongoing project progress and dissemination of multiple research projects. New knowledge was created and shared with academic communities about the impacts of the rapid transition to online learning during the COVID-19 pandemic. Human capital was raised through relationship development.

The dynamics of the research team were impacted by context and input as researchers navigated the obligations and requirements of six research institutions. The research team was successful despite lack of financial resources from the researchers’ institutions and despite a limited history of collaboration among the researchers.

## Discussion

The purpose of this case study was to explore the processes and experiences of members of an interdisciplinary research team. Team members used the Dynamics of Cross-disciplinary Research Development framework to identify the elements that contributed to successful research team science collaboration outputs: Social learning processes, social capital outcomes, and knowledge and human capital outcomes (Carr et al., 2018). In doing so, team members can provide recommendations for experienced and emerging researchers as they engage in the interdisciplinary team science process (Green & Johnson, 2015). Guidance on theories and processes of interdisciplinary research teams is available (Menken & Keestra, 2016; Repko & Szostak, 2020), and the benefits of interdisciplinary teams for productivity and innovation are well documented (Rey-Rocha et al., 2002; Wuchty et al., 2007) but little is known about the experiences of those who organize and sustain health professions education research teams comprised of an interdisciplinary set of scholars. Team members in this study identified transparent team leadership, project management, and shared goals and interests as the factors that led to their success.

The Dynamics of Cross-disciplinary Research Development Conceptual Framework illustrates how interdisciplinary research involves a recursive process where context, social learning processes, social capital outcomes, and knowledge and human capital outcomes occur (Carr et al., 2018). The context in which this research occurred was influenced by a call from an international research organization. Although literature suggested historical collaborations would contribute to interprofessional team success (Borrego & Newswander, 2008) most team members were unknown to each other and had little history of collaboration. However, by engaging in the social learning process, this collaboration led to development of social capital where team members were members of a highly functional interdisciplinary team that produced new knowledge and human capital outcomes as evidenced by multiple publications and conference presentations. This is an example of successful collaboration occurring through the dynamics, interactions, and feedback of diverse team components (Turner & Baker, 2019). This interdisciplinary team used its diversity in conceptualizing and understanding problems, and to create new knowledge about the constructs the team studied as well as about team science itself (Cooksey, 2001). None of the individual team members would have been likely to produce these outcomes as solo researchers or by working within the boundaries of their own discipline.

Team members described their experiences as members of the interdisciplinary research team primarily as positive. As Carr et al. (2018) suggested, values-based research relationships lead to an eagerness to continue working with the team that highlights the recursive process of the framework where research achievements and social learning motivate further collaboration. Challenges suggested in the literature were overcome (Dahlander & McFarland, 2013; Rhoten & Parker, 2004; Zucker, 2012). Early-stage faculty collaborated equally with later-stage faculty and established relationships beyond their own institution. With consideration to career advancement and costs of participation, all team members added publications and presentations to their CVs, with several using the project outcomes and prestige of working with a multi-institutional interdisciplinary team to bolster their promotion dossiers.

## Team science recommendations

Teamwork and collaboration start with transparent and distributed team leadership, as shown in this study, where distributed leadership resulted in decentralization of authority and decision-making across multiple groups. Distributed leadership in this case, was based on the project requirements of reviewing existing literature, designing a survey instrument, and obtaining interinstitutional IRB. Setting ground rules at the start and identifying team members’ motives for joining the team allows the team to coalesce around a shared goal. Identifying team members’ strengths and weaknesses facilitates self-selection onto distributed sub-teams, resulting in knowledge and human capital outcomes where new knowledge is gained, and skills are strengthened.

Team leaders must be responsible for setting timelines and communicating goals and expectations, while all members should be held responsible for adhering to agreed-upon ground rules. It is important that all team members be respectful of the team’s time, knowing that each member has competing priorities. Distributed leadership allows for task delegation where multiple team members can take leadership roles, resulting in shared workload and ownership of project inputs and outputs. This approach allows for more collaboration and shared responsibility, reducing power imbalances and fostering a more inclusive, participatory environment, leading to the social learning, shared understanding, and interpersonal connectivity of social capital outcomes.

Although distributed leadership can lead to strong collaboration, team members must exhibit humility and be open to the ideas and actions of team members with varying levels of experience, and they must be able to respectfully ask questions and challenge one another. Being open to feedback and others’ ideas while engaging in the process can lead to social capital outcomes including future professional collaborations as well as development of professional friendships where resources are shared beyond the research project. In addition, team members must be open to creating new knowledge that comes from their diverse experiential and institutional backgrounds. This can sometimes slow the process down or highlight an unresolved disagreement, but the end result is the creation of new knowledge.

Interdisciplinary research team collaboration comes with challenges (McMurtry et al., 2012), and the findings of this study affirmed the importance of addressing them proactively. Although it can take time, the importance of developing a common mission and shared processes cannot be overstated. A willingness to respectfully work through difficulty or conflict can result in shared learning. Balancing external demands and the contexts of multiple organizations is required. Therefore, each team member must explore their motivations, strengths, weaknesses, and possible barriers as they commit to engagement in team science.

Finally, team members must prepare for the challenges of interinstitutional IRB approval. Researchers on this team faced challenges as each institution had different requirements. Initial IRB approval was received from one institution and was used to request IRB at other institutions. Some institutions required additional review, while others required none. Taking time to understand the approval process within the context of each participating institution can lead to a smoother approval process.

## Limitations

This study provides valuable insight into the experiences of interdisciplinary team members, but there are several limitations to this work. This study used a case study methodology where all team members also served as participants and authors. This approach allows for nuanced accounts and high levels of reflexivity, but it has been criticized for its limited generalizability, concerns of confidentiality in shared settings, and rigor (Tarisayi, 2023). The use of team members as participants may also result in acquiescence bias, with some participants wishing to respond positively to reaffirm the positive outcomes of the group. Concerns for rigor were mitigated by the convergence of data which led to an enhanced understanding of the experiences of the research team providing a blueprint for future interdisciplinary researchers. Parallel data collection methods where data could easily be compared were not used; however, the use of the framework identified areas of data convergence. Limitations of data sources and team composition should be considered. Meeting minutes did not include individual team member content other than attendance, leading to unequal sample sizes between reflection data and meeting minutes data. While the team was professionally and racially diverse, and diverse in areas of research expertise, the team was comprised of US based, female researchers. Future research should utilize external researchers to explore the experiences of teams with diverse identities who are from outside the US and should consider diversity beyond demographic differences.

ChatGPT was used as a “third coder” for the purposes of confirmability after traditional qualitative analysis was complete. Although this approach is innovative, it requires careful scrutiny by the researchers to ensure outputs are factually accurate, aligned with the sound reasoning of the research purpose, and not overly simplified. Generative AI models lack the deep contextual understanding of the topic that human researchers rely on when analyzing qualitative data, and they may overgeneralize and provide biased output. Using generative AI is not a substitute for human coding. Instead, researchers should use generative AI as a supplement to human researcher coding, while maintaining reflexive practice. Finally, researchers must gain IRB approval and, if deemed necessary, participant consent for the use of generative AI in coding.

The need for large research teams has become increasingly evident as the complexity and size of scientific problems have grown not only in the social and behavioral sciences but also in the biological and physical sciences; however, it is not clear that institutional support systems, and individual scientists are evolving with the needs of these science teams (Jackson, 2019). Researchers of all levels and disciplines can use the findings of this study as they identify scholarly opportunities and collaborate with colleagues from different disciplines who will challenge them intellectually. Collaborative research practice and team science have developed in parallel rather than together even though team science is often interprofessional and/or interdisciplinary (Little et al., 2017). Although team science has been explored in medical science, engineering, and other hard sciences, very little research about educational team science exists. In addition, training needs to be adapted for team based and interdisciplinary based researchers of all disciplines (Jackson, 2019; Little et al., 2017). Exploring the experiences of an interdisciplinary team of educational researchers using the Dynamics of Cross-disciplinary Research Development Conceptual Framework provides a template for forming and leading team science projects across disciplines and institutions that can be used to educate future team science researchers.

## Supplementary Information

Below is the link to the electronic supplementary material.

Supplementary file1 (DOCX 15 KB)

## Data Availability

Collected with consent and stored in a protected environment. Correspond with lead author for additional details.

## References

[CR1] Albert, M., Friesen, F., Rowland, P., & Laberge, S. (2020a). Problematizing assumptions about interdisciplinary research: Implications for health professions education research. *Advances in Health Sciences Education,**25*, 755–767. 10.1007/s10459-019-09911-7 31432302 10.1007/s10459-019-09911-7 PMC7359156

[CR2] Albert, M., Rowland, P., Friesen, F., & Laberge, S. (2020b). Interdisciplinarity in medical education research: Myth and reality. *Advances in Health Sciences Education,**25*, 1243–1253. 10.1007/s10459-020-09977-8 32583329 10.1007/s10459-020-09977-8 PMC7704507

[CR3] Borrego, M., & Newswander, L. K. (2008). Characteristics of successful cross-disciplinary engineering education collaborations. *Journal of Engineering Education,**97*(2), 123–134. 10.1002/j.2168-9830.2008.tb00962.x

[CR4] Burbage, A. K., Jia, Y., & Hoang, T. (2022). Community of inquiry, self-efficacy, and student attitudes in sustained remote health professions learning environments. *BMC Medical Education,**23*(1), 481. 10.1186/s12909-023-04382-2 10.1186/s12909-023-04382-2 PMC10303336 37380947

[CR5] Carr, G., Loucks, D. P., & Blöschl, G. (2018). Gaining insight into interdisciplinary research and education programmes: A framework for evaluation. *Research Policy,**47*(1), 35–48. 10.1016/j.respol.2017.09.010

[CR6] ChatGPT. (2023). What are the common categories across the following input. [Response to user question]. https://openai.com/research/overview

[CR7] Cooksey, R. W. (2001). What is complexity science? A contextually grounded tapestry of systemic dynamism, paradigm diversity, theoretical eclecticism. *Emergence, A Journal of Complexity Issues in Organizations and Management,**3*(1), 77–103.

[CR8] Dahlander, L., & McFarland, D. A. (2013). Ties that last: Tie formation and persistence in research collaborations over time. *Administrative Science Quarterly,**58*(1), 69–110. 10.1177/0001839212474272

[CR9] Fiore, S. M., & Wiltshire, T. J. (2016). Technology as teammate: Examining the role of external cognition in support of team cognitive processes. *Frontiers in Psychology,**7*, 1531. 10.3389/fpsyg.2016.01531 27774074 10.3389/fpsyg.2016.01531 PMC5054015

[CR10] Fulmer, T., & Gaines, M. (2014). Partnering with patients, families, and communities to link interprofessional practice and education. In: Macy Conference, Arlington, VA.

[CR11] Green, B. N., & Johnson, C. D. (2015). Interprofessional collaboration in research, education, and clinical practice: Working together for a better future. *Journal of Chiropractic Education,**29*(1), 1–10. 10.7899/JCE-14-36 25594446 10.7899/JCE-14-36 PMC4360764

[CR12] Hall, K. L., Vogel, A. L., Huang, G. C., Serrano, K. J., Rice, E. L., Tsakraklides, S. P., & Fiore, S. M. (2018). The science of team science: A review of the empirical evidence and research gaps on collaboration in science. *American Psychologist,**73*(4), 532. 10.1037/amp0000319 29792466 10.1037/amp0000319

[CR13] Hibbert, P., Siedlok, F., & Beech, N. (2016). The role of interpretation in learning practices in the context of collaboration. *Academy of Management Learning & Education,**15*(1), 26–44. 10.5465/amle.2014.0004

[CR14] Jackson, J.S. (2019) Forward, in Hall, K.L., Vogel, A.L. & Croyle, R.T. (Eds.), *Strategies for team science success: Handbook of evidence-based principles for cross-disciplinary science and practical lessons learned from health researchers.* Springer.

[CR15] Jeong, S., & Choi, J. Y. (2015). Collaborative research for academic knowledge creation: How team characteristics, motivation, and processes influence research impact. *Science and Public Policy,**42*(4), 460–473. 10.1093/scipol/scu067

[CR16] Jia, Y., Gesing, P., Jun, H.-J., Burbage, A. K., Hoang, T., Kulo, V., Cestone, C., McBrien, S., & Tornwall, J. (2023). Exploring the impacts of learning modality changes: Validation of the learning modality change community of inquiry and self-efficacy scales. *Education and Information Technologies,**28*(2), 1763–1781. 10.1007/s10639-022-11258-3 35967826 10.1007/s10639-022-11258-3 PMC9362111

[CR17] Jun, H., Jia, Y., & Kulo, V. (2024). Investigating factors associated with faculty perspectives on changes in teaching modalities within health professions education programs. *Education and Information Technologies*. 10.1007/s10639-024-13034-x

[CR18] Klein, J. T., & Newll, W. H. (1997). Advancing interdisciplinary studies. In J. L. R. J. G. Gaff (Ed.), *Handbook of the Undergraduate Curriculum: A Comprehensive Guide to Purposes, Structures, Practices, and Change* (pp. 393–415). Jossey-Bass.

[CR19] Krishnan, A. (2009). *What are academic disciplines? Some observations on the disciplinarity vs. interdisciplinarity debate* ESRC National Centre for Research Methods, University of Southampton.

[CR20] Larivière, V., Haustein, S., & Börner, K. (2015). Long-distance interdisciplinarity leads to higher scientific impact. *PLoS ONE*, *10*(3), e0122565. 10.1371/journal.pone.0122565 10.1371/journal.pone.0122565 PMC4379013 25822658

[CR21] Little, M. M., St Hill, C. A., Ware, K. B., Swanoski, M. T., Chapman, S. A., Lutfiyya, M. N., & Cerra, F. B. (2017). Team science as interprofessional collaborative research practice: A systematic review of the science of team science literature. *Journal of Investigative Medicine,**65*(1), 15–22. 10.1136/jim-2016-000216 27619555 10.1136/jim-2016-000216 PMC5284346

[CR22] McMurtry, A., Clarkin, C., Bangou, F., Duplàa, E., MacDonald, C., Ng-A-Fook, N., & Trumpower, D. (2012). Making interdisciplinary collaboration work: Key ideas, a case study and lessons learned. *Alberta Journal of Educational Research,**58*(3), 461–473.

[CR23] Menken, S., & Keestra, M. (2016). An introduction to interdisciplinary research: Theory and practice. *An Introduction to Interdisciplinary Research*, 1–128. Amsterdam University Press

[CR24] Merriam, S. B., & Tisdell, E. J. (2016). *Qualitative research: A guide to design and implementation* (4th ed.). Jossey-Bass.

[CR25] O’Sullivan, P. S., Stoddard, H. A., & Kalishman, S. (2010). Collaborative research in medical education: A discussion of theory and practice. *Medical Education,**44*(12), 1175–1184. 10.1111/j.1365-2923.2010.03768.x 21070341 10.1111/j.1365-2923.2010.03768.x

[CR26] Okhuysen, G. A. (2001). Structuring change: Familiarity and formal interventions in problem-solving groups. *Academy of Management Journal,**44*(4), 794–808. 10.5465/3069416

[CR27] Paletz, S. B., Kim, K. H., Schunn, C. D., Tollinger, I., & Vera, A. (2013). Reuse and recycle: The development of adaptive expertise, routine expertise, and novelty in a large research team. *Applied Cognitive Psychology,**27*(4), 415–428. 10.1002/acp.2928

[CR28] Ponomariov, B. L., & Boardman, P. C. (2010). Influencing scientists’ collaboration and productivity patterns through new institutions: University research centers and scientific and technical human capital. *Research Policy,**39*(5), 613–624. 10.1016/j.respol.2010.02.013

[CR29] Pretty, J. (2003). Social capital and the collective management of resources. *Science,**302*(5652), 1912–1914. 10.1126/science.109084 14671287 10.1126/science.1090847

[CR30] Pyrko, I., Dörfler, V., & Eden, C. (2017). Thinking together: What makes communities of practice work? *Human Relations,**70*(4), 389–409. 10.1177/0018726716661040 28232754 10.1177/0018726716661040 PMC5305036

[CR31] Reed, M. S., Evely, A. C., Cundill, G., Fazey, I., Glass, J., Laing, A., Newig, J., Parrish, B., Prell, C., & Raymond, C. (2010). What is social learning? *Ecology and Society,**15*(4), 1–10.

[CR32] Repko, A. F., & Szostak, R. (2020). *Interdisciplinary research: Process and theory*. Sage publications.

[CR33] Rey-Rocha, J., Martín-Sempere, M., & Garzón, B. (2002). Research productivity of scientists in consolidated vs. non-consolidated teams: The case of Spanish university geologists. *Scientometrics,**55*(1), 137–156. 10.1023/a:1016059222182

[CR34] Rhoten, D., & Parker, A. (2004). Risks and rewards of an interdisciplinary research path. *Policy Forum,**306*(5704), 2046–2046. 10.1126/science.110362 10.1126/science.1103628 15604393

[CR35] Saldaña, J. (2021). *The coding manual for qualitative researchers* (4th ed.). Sage Publications.

[CR36] Sell, K., Hommes, F., Fischer, F., & Arnold, L. (2022). Multi-, inter-, and transdisciplinarity within the public health workforce: A scoping review to assess definitions and applications of concepts. *International Journal of Environmental Research and Public Health,**19*(17), 10902. 10.3390/ijerph191710902 36078616 10.3390/ijerph191710902 PMC9517885

[CR37] Stokols, D., Fuqua, J., Gress, J., Harvey, R., Phillips, K., Baezconde-Garbanati, L., Unger, J., Palmer, P., Clark, M. A., & Colby, S. M. (2003). Evaluating transdisciplinary science. *Nicotine & Tobacco Research,**5*(1), S21–S39. 10.1080/14622200310001625555 14668085 10.1080/14622200310001625555

[CR38] Stokols, D., Misra, S., Moser, R. P., Hall, K. L., & Taylor, B. K. (2008). The ecology of team science: Understanding contextual influences on transdisciplinary collaboration. *American Journal of Preventive Medicine,**35*(2), S96–S115. 10.1016/j.amepre.2008.05.003 18619410 10.1016/j.amepre.2008.05.003

[CR39] Tarisayi, K. S. (2023). Autoethnography as a qualitative methodology: Conceptual foundations, techniques, benefits and limitations. *Encyclopaideia,**27*(67), 53–63.

[CR40] Ten Cate, O. (2021). Health professions education scholarship: The emergence, current status, and future of a discipline in its own right. *FASEB BioAdvances,**3*(7), 510–522. 34258520 10.1096/fba.2021-00011 PMC8255850

[CR41] Turner, J. R., & Baker, R. M. (2019). Complexity theory: An overview with potential applications for the social sciences. *Systems,**7*(1), 4. 10.3390/systems7010004

[CR42] Wuchty, S., Jones, B. F., & Uzzi, B. (2007). The increasing dominance of teams in production of knowledge. *Science,**316*(5827), 1036–1039. 10.1126/science.1136099 17431139 10.1126/science.1136099

[CR43] Yin, R. K. (2018). *Case study research and applications* (Vol. 6). Sage Publications.

[CR44] Zucker, D. (2012). Developing your career in an age of team science. *Journal of Investigative Medicine,**60*(5), 779–784. 10.2310/JIM.0b013e3182508317 22525235 10.231/JIM.0b013e3182508317 PMC3665004

