# Supplementary material for: Team science in interdisciplinary health professions education research: a multi-institutional case study
Source: Adv Health Sci Educ Theory Pract. 2024 Nov 16;30(4):1123–41. doi: 10.1007/s10459-024-10393-5 (PMC12391146; doi:10.1007/s10459-024-10393-5)
Supplement: Supplementary file 1 — Supplementary file1 (DOCX 15 KB) [file 10459_2024_10393_MOESM1_ESM.docx]

**Appendix A**

**Data Collection: Team member reflections**

***Consent***

You are being asked to share your written reflections about your experience as a member of the learning modality changes research team. The team began collaboration May 19, 2021, on research related to learning modality changes during the Covid-19 pandemic.

While engaging in reflection, you will encounter minimal risks, including the possibility that anxiety or unpleasant experiences will surface as a result of your reflections. Your confidentiality will be protected to the maximum extent allowable by law. Any direct identification of information will be removed from data when responses are analyzed. All data will be secured in password protected files and will be accessible only to the two researchers [fname lname and fname lname] tasked with analyzing the data.

By sharing your written reflection, you are consenting to participation in this study.

*Please use this document to complete your reflection. Save and email completed reflection along with your CV to [fname] at [email]*

***Researcher Reflection***

Include the following demographic content that will be viewable only by [fname] and [fname] and used for data analysis.

1. Share your experience level with the following areas using the scale:

None 2) almost none 3) some 4) a moderate amount 5) a great deal

IRB proposal and approval

Quantitative research methods

Survey design

Educational research

Team research

Research team leadership

Interinstitutional research

Interdisciplinary research

1. Reflect on your experience since May 2021 on the interinstitutional/interdisciplinary team project related to learning modality changes during the Covid-19 pandemic. The following prompts are provided to guide your reflection, but you are not required to answer any or all questions.
2. Describe how you came to participate in the research team, how you learned about it.
3. Why did you volunteer to join the research team?
4. What did you hope to gain from this experience?
5. What was it about the team that made you stay with the team long-term?
6. What elements of the project made the project worth engaging in?
7. What challenges did the team face?
8. What challenges (and/or personal barriers) did you face?
9. Did you overcome the barriers, and if so, how?
10. What practical advice would you give to others undertaking a similar initiative?
11. Do you want to continue working with this team?
12. If you answered yes, how?
13. If you answered yes, what role would you like to take on future projects?
14. If you answered no, why not?
15. How has this project affected other aspects of your work?
16. How has this project affected your career progression?
17. How has this project affected your feelings about team research?
18. How did this project change or transform how you would lead or be in a team?
19. What value did you find in being on an interinstitutional/interprofessional team?
20. Other reflections...
